# Home Foreclosure, Health, and Mental Health: A Systematic Review of Individual, Aggregate, and Contextual Associations

**DOI:** 10.1371/journal.pone.0123182

**Published:** 2015-04-07

**Authors:** Alexander C. Tsai

**Affiliations:** 1 Center for Global Health, Massachusetts General Hospital, Boston, Massachusetts, United States of America; 2 Harvard Medical School, Boston, Massachusetts, United States of America; 3 Mbarara University of Science and Technology, Mbarara, Uganda; University of Pennsylvania, UNITED STATES

## Abstract

**Background:**

The U.S. foreclosure crisis intensified markedly during the Great Recession of 2007-09, and currently an estimated five percent of U.S. residential properties are more than 90 days past due or in the process of foreclosure. Yet there has been no systematic assessment of the effects of foreclosure on health and mental health.

**Methods and Findings:**

I applied systematic search terms to PubMed and PsycINFO to identify quantitative or qualitative studies about the relationship between home foreclosure and health or mental health. After screening the titles and abstracts of 930 publications and reviewing the full text of 76 articles, dissertations, and other reports, I identified 42 publications representing 35 unique studies about foreclosure, health, and mental health. The majority of studies (32 [91%]) concluded that foreclosure had adverse effects on health or mental health, while three studies yielded null or mixed findings. Only two studies examined the extent to which foreclosure may have disproportionate impacts on ethnic or racial minority populations.

**Conclusions:**

Home foreclosure adversely affects health and mental health through channels operating at multiple levels: at the individual level, the stress of personally experiencing foreclosure was associated with worsened mental health and adverse health behaviors, which were in turn linked to poorer health status; at the community level, increasing degradation of the neighborhood environment had indirect, cross-level adverse effects on health and mental health. Early intervention may be able to prevent acute economic shocks from eventually developing into the chronic stress of foreclosure, with all of the attendant benefits this implies for health and mental health status. Programs designed to encourage early return of foreclosed properties back into productive use may have similar health and mental health benefits.

## Introduction

At the tail end of the Great Recession (2007–09), during which housing prices were experiencing the steepest decline in U.S. history [1] and there were more than 6 million properties with foreclosure filings (representing between 1–2 percent of all U.S. housing units in any given year) [2], Bennett and colleagues [3] asked the perceptive question: *will the public’s health fall victim to the home foreclosure epidemic*? At the time, they were unaware of any studies specifically examining the effects of home foreclosure on health or mental health. Similarly, in a systematic review of the health effects of economic downturns, Modrek and colleagues [4] also identified the need for more research to understand how foreclosures and other aspects of housing insecurity undermine health. Since the end of the Great Recession, there have been more than 8 million additional properties with foreclosure filings in the U.S. [2]. Yet, although a crisis on this scale is likely to have (or have had) significant adverse impacts on population health and mental health, to date there has been no summative assessment of this literature.

At the level of the individual, prior research yields simple predictions about the associations between foreclosure, health, and mental health. Even though Bennett and colleagues [3] had not, at the time of their writing, identified any studies specifically supporting an adverse effect of foreclosure, reasonable extrapolation from related studies would tend to support this expectation. There is an extensive literature on the adverse health effects of related stressful life events [5,6], economic strains such as job loss [7] and personal unsecured debt [8]. Conversely, the salubrious effects of housing and homeownership are well known [9,10]. These related lines of inquiry suggest that the personal experience of foreclosure would be expected to have adverse impacts on individual health and mental health.

The experience of home foreclosure differs from other stressful life events and/or economic strains in several different ways. First, homeownership uniquely buttresses self-identity in a way that other assets and economic activities do not [11], thereby providing, as described by Saunders [12], “ontological security.” Second, the experience of home foreclosure is rarely a discrete, time-limited event [13]. Before delinquent borrowers are served with a legal notice of foreclosure, they may be able to catch up on payments, modify or refinance their mortgages, or even sell their properties. The same options are available after the legal notice of foreclosure is received, or the property may remain in limbo. Ultimately the lender may elect to force a foreclosure auction, or the property may become real-estate owned. Thus, foreclosure frequently involves stress of protracted duration. Third, foreclosure may involve relocation and disruption of existing social networks [14,15]. And finally, the adverse effects of foreclosure do not occur in an historical vacuum and may uniquely worsen racial or ethnic disparities in health [16,17]. Therefore, it is probable that home foreclosure represents a unique economic strain with concomitantly greater adverse impacts on health and mental health than other types of strains.

Mathematically, testing this hypothesis at the individual level can be illustrated with the stylized equation below. At the individual level,

$$y_{i}=\alpha x_{i}+\delta z_{i}+e_{i}$$ (1)

where *y*_*i*_ represents the health (or mental health) status of individual *i*; *x*_*i*_ represents the individual’s personal experience of home foreclosure; z_i_ represents additional covariates measured at the individual level, such as potential confounders; and *e*_*i*_ represents residual differences in individual health status after accounting for the personal experience of home foreclosure.

A similar equation can be written to test this hypothesis at the aggregate level,

$$Y_{j}=\beta X_{j}+\gamma Z_{j}+u_{j}$$ (2)

where *Y*_*j*_ represents the average health status of a cluster *j* of individuals (e.g., grouped together in a county or state); *X*_*j*_ represents the home foreclosure rate in cluster *j*; Z_j_ represents additional covariates measured at the cluster level, such as potential confounders; and *u*_*j*_ represents residual differences in cluster-level health status after accounting for the home foreclosure rate. However, analyses of the relationship between foreclosure and health outcomes based on aggregate-level data may be subject to the ecological fallacy, as noted by Houle and Light [18] in their analysis of state-level data on the association between rates of foreclosure and rates of suicide.

Multilevel data are particularly well suited to understanding the *contextual* effect of home foreclosure on health or mental health. That is, the local home foreclosure environment could compromise individual health or mental health status apart from any personal experience of home foreclosure (e.g., through a stress-mediated process). Such a study would require data with exposures specified at different levels, with personal experience of home foreclosure measured at the individual level and the local rate of home foreclosures measured at the aggregate level, similar to the study by Tapia Granados and colleagues [19] in which unemployment measured at both the individual and the contextual level were measured. Accordingly, testing for the contextual effect of home foreclosure on health would require a model such as

$$y_{ij}=\alpha x_{ij}+\beta X_{j}+\delta z_{ij}+\gamma Z_{j}+u_{j}+e_{ij}$$ (3)

where *y*_*ij*_ represents the health status of individual *i* living in cluster *j*; *x*_*ij*_ represents the personal experience of home foreclosure for individual *i* living in cluster *j*; X_j_ represents the home foreclosure rate in cluster *j*; *z*_*ij*_ represents additional covariates for individual *i* living in cluster *j*; *Z*_*j*_ represents additional covariates measured at the cluster level; and *u*_*i*_ and *e*_*i*_ representing residual differences as noted. Importantly, if there is a substantive interest in understanding the stress-mediated effects of the local foreclosure environment, it may be important to include *x*_*ij*_ as a potential confounder [20].

Taken together, these conceptual considerations and findings from related studies provide an evidence basis for concern about the potential public health and mental health fallout resulting from the foreclosure crisis. To address this important gap in the literature, I undertook this systematic review with the aim of understanding the channels through which home foreclosures adversely affect health and mental health.

## Methods

### Ethics Statement

All study procedures were reviewed by the Partners Human Research Committee and deemed exempt from full review because the study was based on anonymous, public-use data with no identifiable information on participants.

### Systematic Evidence Search

This systematic review was conducted according to the Preferred Reporting Items for Systematic Reviews and Meta-Analyses (PRISMA) guidelines (S1 Checklist). The systematic evidence search was conducted in June 2014. Two bibliographic databases were used, PubMed and PsycINFO (specific search terms described in S1 Table). After all citations were imported into EndNote reference management software (version X6, Thomson Reuters, New York, NY), the “Find Duplicates” algorithm was used to exclude duplicate references. The titles and abstracts, and then the full texts of the articles, were sequentially screened to select articles for inclusion. To identify other potentially relevant studies, I searched the reference lists of included articles and queried other colleagues in the field.

Selected articles had to have involved a quantitative or qualitative analysis of the relationship between home foreclosure and health or mental health. Therefore, I excluded studies about the health effects of general economic crises, personal unsecured debt, chronic homelessness (unless the study’s focus was on homelessness related to home foreclosure), poor housing conditions, and loss of housing due to natural disasters, as all of these stressors have previously been subject to detailed study [8,21,22,23,24,25,26,27]. I also excluded studies focused exclusively on earlier segments of the foreclosure process, such as mortgage strain or overall indebtedness or housing unaffordability. Finally, the exclusion criteria also resulted in the exclusion of studies, primarily from the sociological literature, about foreclosure, neighborhood degradation, and crime.

To assess the quality of the included quantitative studies, I developed an assessment tool based on major conceptual domains identified by Sanderson and colleagues [28]. The criteria I used were as follows: (1) the study was based on a population-based sample of participants; (2) the study employed an objective measure of exposure to foreclosure; (3) the study employed an objectively measured health or mental health outcome; (4) the exposure preceded the outcome; and (5) the analysis adopted a strategy that could account for unobserved confounding.

### Data Analysis

For each selected article, data were extracted regarding the study design, population, sample size, foreclosure- and health-related variables, and conclusions. Overlapping analyses of the same dataset were aggregated and treated as a single study. To assess the extent to which the field has addressed two priority areas for research that were highlighted by Bennett and colleagues [3], I also determined whether or not the studies based on individual-level data investigated (a) the interaction between home foreclosure and other types of stressful life events (e.g., job loss) or chronic strains, and (b) differential adverse effects on racial and ethnic minorities. Due to substantial methodological differences in study designs and the exposures and outcomes assessed, I did not attempt to summarize the data using meta-analysis.

## Results

### Studies Identified for this Review

**Fig 1:**
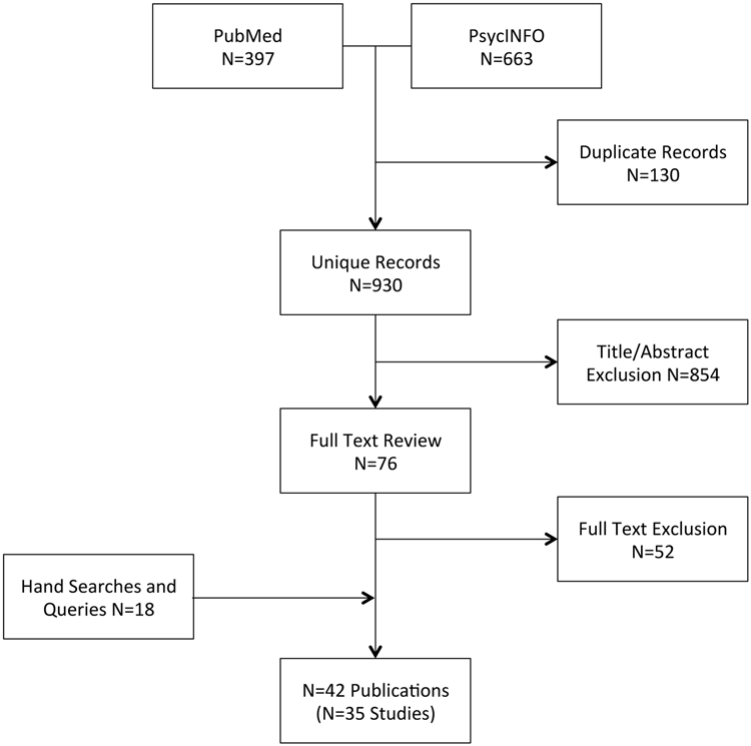
Preferred Reporting Items for Systematic Reviews and Meta-Analyses (PRISMA) flow diagram depicting the number of publications screened and included in the systematic review.

The PsycINFO search yielded 663 records, while the PubMed search yielded 397 records (Fig 1). Of these, 103 were identified as duplicates using automated software; another 27 were manually identified as duplicates (e.g., alternate spellings of author names, dissertations matched to subsequent peer-reviewed journal articles, articles reprinted as book chapters, etc.). I screened the titles and abstracts of the remaining 930 publications and reviewed the full text of 76 articles, dissertations, and other reports. Ultimately, I included 24 records representing 21 unique studies. Hand searches of reference lists yielded another 10 articles representing six additional unique studies. Queries of colleagues yielded an additional six unique studies, including one study from the personnel psychology literature, one unpublished study from the economics literature, three studies that were published after the systematic search was completed, and one study that was not captured through the systematic search. A peer reviewer suggested an additional two studies that were not captured through the systematic search. Therefore a total of 42 publications representing 35 unique studies were included in this review (Table 1). The number of publications exceeds the number of unique studies because in several instances a single study generated multiple publications.

**Table 1:** Peer-Reviewed Journal Articles and Other Publications Describing the Associations between Foreclosure, Health, and Mental Health.

Study	Years of Data Collection	Study Design	Foreclosure Variable	Health-Related Outcome	Conclusions
Arcaya and colleagues [77, 78]	1987–2008	Cohort of 2,068 participants in the U.S. Framingham Offspring Cohort Study	Number of real-estate owned foreclosures within 100 meters	Body mass index (BMI) and physician-assessed sitting systolic blood pressure	Each additional foreclosed property within 100 meters was associated with a 0.2-unit difference in BMI and a 1.71 mm/Hg difference in SBP
Ayers and colleagues [79]	2004–10	Monthly time series analysis of aggregated Google search engine queries	Proportion of conventional single-family loans 90 days past due or in the foreclosure process, averaged from Fannie Mae and Freddie Mac	Aggregate volume of search queries related to psychological distress	A one-percentage point increase in mortgages in delinquencies and foreclosure was associated with a 16 percentage point increase in volume of psychological distress queries the following month
Batson and Monnat [80]	2009	Cross-sectional survey of persons in 643 households in Clark County, Nevada	Rate of foreclosure in the census tract in 2007 (matched to 22 distinct neighborhoods), as estimated by the Department of Housing and Urban Development using data obtained through the Neighborhood Stabilization Program	Quality of life	The census tract-level foreclosure rate had a statistically significant negative association with quality of life (b = -0.11; s.e. = 0.04).
Brooks-Gunn and colleagues [81]	2007–10	Cross-sectional analysis of data from 2,032 women participating in the 9-year follow-up of the U.S. Fragile Families and Child Wellbeing Study	Mortgage foreclosure rate in the study participant’s state at the time (quarter) of interview	Frequency of child spanking, based on the Conflict Tactics Scale for Parent and Child	The state-level foreclosure rate did not have a statistically significant association with child spanking (data not shown)
Burgard and colleagues [82]	2009–10	Stratified random sample of 894 non-institutionalized, English-speaking adults in southeastern Michigan	Being behind on mortgage payments or in the foreclosure process (among study participants currently paying a mortgage) and foreclosure after 2007 (among study participants who had ever owned a home)	Poor or fair self-rated health, positive depression screen on the nine-item Patient Health Questionnaire (PHQ-9), anxiety attack within the four weeks prior to interview, and positive screen for harmful alcohol use on the 10-item Alcohol Use Disorders Identification Test	Being behind on mortgage payments or in the foreclosure process, or having experienced a recent foreclosure, had statistically significant associations with fair/poor self-rated health, positive screen for depression, and recent self-reported anxiety attack (adjusted odds ratios [AORs] ranged from 3.09–5.76) but not with harmful alcohol use
Cagney and colleagues [83]	2005–11	Cohort of 1,883 older adults participating in the U.S. National Social Life, Health, and Aging Project who did not have significant depressive symptoms at the baseline interview	Large increase (i.e., greater than median value across all zip codes) in proportion of housing units within the study participant’s zip code that received notices of default, went to auction, or were real-estate owned	Significant depressive symptoms according to the 11-item Iowa Short Form of the Centers for Epidemiologic Studies Depression scale (CES-D)	Development of depressive symptoms had statistically significant associations with large increases in the proportion of housing stock receiving notices of default or that were real estate-owned (AORs ranged from 1.62–1.75) but not with the proportion of housing stock that went to auction
Cannuscio and colleagues [84]	2008	Cross-sectional survey of 798 adults living in Arizona, California, Florida, and Nevada who belonged to the volunteer participant panel of an Internet survey research company	Being more than 30 days behind on mortgage payments, having received a notice of default or foreclosure from a lender, and/or having experienced loss of a home through foreclosure in the previous 12 months	Poor or fair self-rated health, health compared to 12 months previous, serious psychological distress on the K6 scale, experience of 12 different somatic symptoms	Being in default or foreclosure was associated with worse self-rated health and serious psychological distress (AORs ranged from 2.14–13.6) and with 10 of 12 somatic symptoms (AORs ranged from 1.12–1.95)
Collier-Goubil [85]	2003–09	Cross-sectional analysis of data from 373 neighborhoods (census block groups) in Mecklenburg County, North Carolina	Rate of completed foreclosures	Rate of citizen-initiated calls to police department involving domestic violence	Each additional neighborhood foreclosure (per 1,000 households) was associated with 2.77 more neighborhood service calls about domestic violence (P<0.001)
Cook and Davis [65]	2000–05	Case-control study of all 315 adult suicides in an urban county in Ohio; a control group of 630 age-, sex-, race-, and geography-matched non-injury deaths; and a second control group of 630 age-, sex-, race-, and geography-matched unintentional injury and poisoning deaths	Experience of a civil court case involving foreclosure within one year prior to death	Completed suicide	A greater proportion of suicide victims experienced foreclosure in the year before death compared to persons who died due to non-injury causes (odds ratio [OR], 3.0; 95% confidence interval [CI], 1.2–7.7) and compared to unintentional injury and poisoning deaths (OR, 3.6; 95% CI, 1.5–8.5)
Currie and Tekin [86]	2005–09	Longitudinal analysis of quarterly data from 3,525 zip codes in Arizona, California, Florida, and New Jersey	Rate of foreclosures (housing units that received a notice of trustee sale and/or notice of foreclosure sale) and volume of real-estate owned properties in the previous quarter	Rates of emergency department (ED) visits and inpatient hospitalizations (in the aggregate as well as those associated with prevention quality indicators)	In regression models with county, quarter, and year fixed effects, 100 additional foreclosures are associated with a 1.2 percent increase in ED visits and hospitalizations, a 4.9–6.7 percent increase in preventable hospitalizations among the non-elderly, a 12.0–18.8 percent increase in ED visits or hospitalizations related to anxiety, a 38.5–41.7 percent increase in ED visits or hospitalizations for suicide attempts
Ertl [87]	2011 *	Qualitative interviews with six adolescents and four primary caregivers in Massachusetts whose families had experienced foreclosure within the previous five years	N/A	N/A	Worry, self-blame, and sadness were themes commonly voiced by study participants, less so among the adolescents compared to their caregivers
Fields and colleagues [31], Libman and colleagues [32]	2006	Nine focus-group and two individual interviews with 88 homeowners in New York City, New York; St. Louis, Missouri; Hamilton, Ohio; Duluth, Georgia; and Waco, Texas who had sought foreclosure intervention services or who had missed payments on an outstanding loan	N/A	N/A	Stress, anxiety, and depression were common themes discussed in all focus-group interviews
Fowler and colleagues [88]	2005–10	Analysis of all 929 eviction- or foreclosure-related suicides documented in 16 states participating in the National Violent Death Reporting System	Loss of housing or impending loss of housing, identified on the basis of key words such as “evicted,” “sheriff sale or warrant of removal,” “foreclosure,” or “lost house or home”	Suicide	The number of suicides related to eviction or foreclosure increased from 88 in 2005 to 176 in 2010
Freeman and colleagues [89]	2008 *	Cross-sectional survey of a stratified random sample of 3,000 farm households in Iowa, with a 45% response rate	Foreclosure on a mortgage or loan	Stress rating, on a scale from 0–100	Foreclosure on a mortgage or loan was ranked as the fifth most stressful life event (mean score, 72.7), behind death of a spouse/child and disabling injury to self/family
Frioux and colleagues [90]	2000–10	Longitudinal analysis of annual data from 67 counties in Pennsylvania	Rate of foreclosures	Count of child maltreatment reports investigated by county child protective services (CPS), and count of substantiated cases of child maltreatment	A 1 percentage point increase in the foreclosure rate was associated with a 3.9 percent increase in CPS investigations (P = 0.04) and a 4.5 percent increase in substantiated cases (P = 0.01)
Gili and colleagues [91]	2010–11	Cross-sectional survey of 5,876 persons seen in primary care visits throughout Spain	Difficulty repaying mortgage loans, housing foreclosure	Major depressive disorder	Having a diagnosis of major depressive disorder had statistically significant associations with housing foreclosure (AOR, 2.95, P<0.001) and housing repayment difficulties (AOR, 2.12, P<0.001)
Houle [92]	2006–11	Longitudinal analysis of annual individual-level data linked to 2,245 counties in the U.S.	Proportion of mortgaged housing units in any stage of the foreclosure process and proportion that are real-estate owned	Single-item measure of mental health: “**T**hinking about your mental health, which includes stress, depression and problems with emotions, for how many days during the past 30 days was your mental health not good?””	Each 0.01 increase in the proportion of real-estate owned properties was associated with a 0.066 increase in the number of poor mental health days, with stronger associations observed in counties with the highest proportions of African Americans
Houle and Light [18]	2005–10	Longitudinal analysis of annual data from all 50 states and Washington, D.C.	Proportion of mortgaged housing units in any stage of the foreclosure process and proportion that are real-estate owned	Age-adjusted suicide rate	Each 0.01 increase in the proportion of foreclosed and real-estate owned properties was associated with a 0.04–0.16 increase in the within-state suicide rate, with the most pronounced associations among the middle-aged and near-elderly
Keene and colleagues [30]	2012–13	Qualitative interviews with 28 adults living in a pseudonymous northeastern U.S. city, who were experiencing mortgage strain	N/A	N/A	Poor health and unanticipated medical bills were a common theme voiced in many interviews; these exacerbated mortgage strain in the context of fragile personal and public safety nets that could not be relied upon to buffer against the adverse effects of illness
McLaughlin and colleagues [93]	2008–10	Cohort of 1,547 predominately African-American participants in the U.S. Detroit Neighborhood Health Study	Experience of home repossession by a creditor due to non-payment within the previous two years	Symptoms of major depressive disorder (PHQ-9) and symptoms of generalized anxiety disorder (7-item generalized anxiety disorder scale)	Experience of foreclosure in the previous two years had statistically significant associations with symptoms of major depressive disorder (incidence rate ratio [IRR], 2.4; 95% CI, 1.59–3.64) and generalized anxiety disorder (IRR, 1.9; 95% CI, 1.36–2.62)
Menzel and colleagues [94]	2005–08	Longitudinal analysis of data collected from patients with one of 25 diagnostic-related groups discharged from non-federal hospitals in Clark County, Nevada	None indicated	Rate of discharges with a principal diagnosis of bipolar or depressive disorder, expressed as a percentage of all discharges	The rate of bipolar or depressive disorder discharges increased in 2007, coinciding with the increase in foreclosure filings observed nationwide.
Mulia and colleagues [95], Zemore and colleagues [96], Murphy and colleagues [97]	2009–10	Cross-sectional survey of 5,382 adult participants in the U.S. National Alcohol Survey	Loss of housing, either owned or rented, between 2008 and the date of the interview	Graduated frequency alcohol volume, drunkenness on a monthly or more frequent basis, experience of 2+ negative consequences related to drinking, and alcohol dependence	Housing loss had a statistically significant positive association with negative drinking consequences (AOR, 9.52; 95% CI, 3.69–24.6) and alcohol dependence (AOR, 5.93; 95% CI, 2.07–17.0) and a null association with alcohol volume and monthly drunkenness
Nettleton and Burrows [98, 99]	1998	Qualitative interviews with 44 adults and 17 children/young people from 30 families who had experienced mortgage repossession	N/A	N/A	All study participants described repossession as a stressful and isolating experience due to the uncertainty, emotional intensity, loss of control, and shame, with concomitant adverse impacts on health behaviors and chronic conditions
Osypuk and colleagues [100]	2009–10	Cohort of 662 participants in the Life-course Influences on Fetal Environments Study of African-American women aged 18–45 years who had recently given birth to a singleton baby in Detroit, Michigan	Experience of foreclosure during pregnancy or in the two years prior to giving birth	Severe depressive symptoms on the 20-item CES-D, with a cutoff of ≥23	Foreclosure was associated with a 4.04-point increase in depressive symptoms (95% CI, 1.24–6.84), or 0.4 standard deviation units, and an increased risk of severe depressive symptoms (adjusted risk ratio [ARR], 1.76; 95% CI, 1.25–2.47)
Pence and colleagues [101], Mugavero and colleagues [102]	2001–04	Cohort of 611 participants in the Coping with HIV/AIDS in the Southeast Study of persons living with HIV receiving medical care in Alabama, Georgia, Louisiana, North Carolina, and South Carolina	Experience of a severe stressor in the previous 27 months, including divorce/ separation, death or illness of an immediate family member, major financial problems (e.g. foreclosure), spending more than 1 week in prison, and/or sexual or physical assault	Self-reported unprotected anal or vaginal penetrative sexual intercourse with an HIV-negative partner or partner of unknown serostatus; and (among 474 persons on HIV treatment at baseline) HIV treatment non-adherence and virologic failure	Experience of a severe stressor had a statistically significant association with virologic failure (AOR, 1.19; 95% CI, 1.02–139) but not unprotected intercourse (AOR, 0.98; 95% CI, 0.68–1.42) or treatment non-adherence (AOR, 1.13; 95% CI, 0.99–1.29); when disaggregated by type, experience of a financial stressor (including foreclosure, car repossession, and other major financial problems) did not have a statistically significant association with non-adherence (AOR, 1.05; 95% CI, 0.78–1.41) or virologic failure (AOR, 0.92; 95% CI, 0.66–1.29)
Pevalin [103]	1991–2008	Longitudinal analysis of annual data from 12,390 participants in the British Household Panel Survey	Experience of repossession or eviction in the past year	Positive screen for common mental illness on the 12-item General Health Questionnaire, with a cutoff of ≥4	Common mental illness had a statistically significant association with repossession (AOR, 1.61; 95% CI, 1.10–2.36) but not eviction (AOR, 0.97; 95% CI, 0.76–1.20)
Pollack and colleagues [104]	2005–08	Case-control study of 404 adults who had experienced foreclosure and who could be matched to at least one primary care appointment within a specific university health system in the U.S.; and a control group of 2,020 zip code-matched adults receiving primary care in the same system.	Experience of foreclosure in 2005–08	Charlson Comorbidity Index, prevalence of 14 specific comorbidities, and health care utilization in the two years prior to foreclosure	Persons who had experienced foreclosure had a higher prevalence of hypertension and renal disease, and a higher Charlson Comorbidity Index; and were more likely to have had an outpatient appointment or to have visited the emergency department
Pollack and Lynch [29]	2008	Cross-sectional survey of 250 Philadelphia residents undergoing judicial foreclosure proceedings, and 10,007 adults participating in the Southeastern Pennsylvania household Health Survey	Experience of foreclosure proceedings	Poor or fair self-rated health, prevalence of six different clinician-diagnosed chronic conditions, health care utilization, and smoking status	Experience of foreclosure had statistically significant associations with hypertension, heart disease, psychiatric conditions, lack of health insurance, cost-related unmet health needs, and cost-related prescription non-adherence (AORs ranged from 1.67–3.44) but not self-rated health, diabetes, asthma, arthritis, emergency department utilization, or smoking status
Ragins and colleagues [105]	2009	Cross-sectional survey of 2,135 alumni of a large midwestern U.S. university who were organizationally employed homeowners	3-item scale measuring fear of home foreclosure	8-item Somatic Complaints at Work Scale	Fear of home foreclosure had a statistically significant positive association with physical symptoms of stress at work; the authors concluded the association was fully mediated by negative home-to-work spillover
Reisen and colleagues [106, 107]	2007	Cross-sectional analysis of 140 laboratory-confirmed human cases of West Nile Virus in Bakersfield, California	N/A	N/A	The authors concluded that the outbreak of West Nile Virus cases was likely caused by a concomitant increase in the number of abandoned neglected swimming pools associated with foreclosed homes
Ross and Squires [108]	2008–09	Qualitative interviews with 22 adults throughout the U.S. who either had experienced foreclosure or were at different stages of mortgage delinquency	N/A	N/A	Themes voiced by all study participants included anxiety and depression, while many participants described feelings of shame and embarrassment
Schootman and colleagues [109]	2007–09	Cross-sectional survey of 1,047 women in Missouri with a new diagnosis of a primary breast cancer	Housing and Urban Development agency foreclosure-abandonment score at the census tract level	Poor or fair self-rated health	In the regression model with the best fit to the data, living in a census tract of high foreclosure risk did not have a statistically significant association with self-rated health (AOR, 1.15; 95% CI, 0.62–2.13)
Scully and colleagues [110]	1997	Cross-sectional survey of a random digit dialing sample of 370 adult residents in Florida, with a 54% response rate	N/A	N/A	Foreclosure on a mortgage or loan was ranked as the 11th most stressful life event (mean score, 36)
Stack and Wasserman [111]	1997–2000	Detailed qualitative assessment of 62 decedents in the U.S. whose suicides occurred in the context of economic strain	N/A	N/A	Most suicides (43/62) occurred in the context of two or more different strains, most commonly economic plus relationship strain (20/43) and two combined economic strains (16/43); of the latter, the most common pattern was housing loss plus unemployment (4/16)
Wood and colleagues [112]	2000–09	Longitudinal analysis of monthly discharge data from 43 major freestanding children’s hospitals in the U.S.	Rates of mortgage foreclosure and 90-day mortgage delinquency in the MSA	Rates of hospitalizations for physical abuse among children <6 years of age and hospitalizations for high-risk traumatic brain injury (TBI) among infants <12 months of age	Each 1 percentage point increase in 90-day delinquency was associated with a 3.1 percent relative increase (95% CI, 0.93–5.30) in the rate of physical abuse and a 4.8 percent relative increase (95% CI, 2.66–7.07) in the rate of high-risk TBI, while each 1 percentage point increase in mortgage foreclosure was associated with a 6.5 percent increase (95% CI, 1.69–11.55) in physical abuse and a 10.2 percent increase (95% CI, 5.56–15.1) in high-risk TBI

* Date of data collection not explicitly noted in the publication and was therefore imputed using the year of publication.

Nearly all studies (32 [91%]) were based on data collected in the U.S., and most studies had data collection periods that overlapped at least partially with one or more years spanning the Great Recession of 2007–09 (26 [74%]). Among the nine studies that did not partially coincide with the Great Recession, six were completed prior to 2007 and three were initiated after 2009. Thirteen studies were based on longitudinal data (37%), 14 (40%) were cross-sectional, six (17%) were qualitative, and two (6%) were case-control. In terms of the unit of analysis, eight studies (23%) were based on data collected at the aggregate level (e.g., census tracts or neighborhood units), while 27 studies (77%) were based on individual-level data. The studies based on individual-level data enrolled a total of 2,646,247 participants, with a median sample size of 1,047 (interquartile range, 200–2,135). Five studies were based on multilevel data.

### Associations between Foreclosure and Mental Health, Health Behaviors, and Health

Most studies concluded (32 [91%]) that foreclosure had adverse effects on the outcomes studied, while three studies (9%) yielded null or mixed findings. Among these 32 studies, most examined a mental health related outcome (24/32 [75%]), while fewer examined outcomes related to physical health (10/32 [31%]), health behaviors (4/32 [13%]), or domestic violence or child abuse (3/32 [9%]). Three of the four studies that examined a health behavior outcome focused on substance abuse, so these were grouped together with the mental health studies in the subsequent discussion for ease of exposition. The three studies with null or mixed findings were all based on individual-level data: two examined a health outcome, while one examined a child abuse outcome.

In all studies that examined an outcome related to mental health or health behavior (including substance use) (25/25 [100%]), foreclosure was associated with worsened outcomes. Among all 21 studies based on individual-level data, the personal experience of home foreclosure was associated with worsened outcomes including depression, anxiety, alcohol use, psychological distress, and suicide. Notably, only two of these studies accounted for potential selection into foreclosure by adjusting for baseline (pre-foreclosure) comorbidity. The estimated associations in the 4 studies based on aggregate-level data mirrored those of the individual-level studies, except that the outcomes studied were (for example) state-level suicide rates instead of personal suicide risk. Finally, similar themes of stress, anxiety, depression, and shame were almost universally described by study participants in the six qualitative studies.

Among the health-related studies, most (10/12 [83%]) showed that foreclosure was associated with worsened outcomes. Among the 10 studies based on individual-level data, in eight studies (8/10 [80%]) the personal experience of foreclosure was associated with worsened health outcomes. Most of these outcomes were self-reported, such as self-rated health, with the exception of one study that determined body mass index and sitting systolic blood pressure through physical examination. The estimated associations in the two studies based on aggregate-level data mirrored those of the individual-level studies. Themes related to poor self-rated health, unhealthy behaviors like cigarette and alcohol use, and financial tradeoffs resulting in unmet medical needs were also commonly voiced by participants in the qualitative studies.

### Mediators and Moderators

Among the five studies based on multilevel data correlated aggregate-level exposures (e.g., county-level foreclosure rate) with individual-level outcomes (e.g., depression), most (3/5 [60%]) yielded a statistically significant association, suggesting that foreclosure at the community level could be linked to individual-level outcomes through mechanisms such as reduced neighborhood-based physical activity or stress-coping responses resulting from degradation of the neighborhood environment. Importantly, none of these studies adjusted for personal experience of foreclosure, which Diez Roux [20] identified as both an important source of foreclosure-related stressor and also a potential confounder of the stress-mediated effects of nearby foreclosures. Non-stress pathways may be possible. One unique study attributed an outbreak of West Nile Virus to the expanding number of neglected swimming pools and Jacuzzis attached to foreclosed properties, suggesting that in some instances foreclosure may have direct impacts on health status.

Eight studies (8/35 [23%]) assessed for interactions between foreclosure and other factor. Among the seven studies based on individual-level data, the personal experience of foreclosure interacted with other types of stressful life events or chronic strains, with concomitant magnified impacts on health and mental health. One study based on aggregate-level data examined the extent to which the association between foreclosure rates and domestic violence rates was greater in economically strained neighborhoods, but failed to detect a statistically significant product term. Only two studies (2/35 [6%]) examined differential adverse effects on racial and ethnic minorities.

### Risk of Bias

All but three of the quantitative studies were judged to be at potential risk of bias (S2 Table). While many studies were based on population samples, had objectively measured exposure and outcome data, or ensured a temporal ordering between the exposure and outcome, few studies were able to account for unobserved confounding. No experimental or quasi-experimental studies were identified in the systematic evidence search. (While an experiment in which study participants were randomized to foreclosure is unlikely to ever be conducted for obvious reasons, it is conceivable to imagine an anti-foreclosure intervention study in which health and mental health outcomes were measured as part of the study design.) Of the 13 longitudinal studies, only six employed a statistical analysis that exploited within-unit variation over time to adjust for potential confounding by observed and/or unobserved time-invariant factors.

Of note, while most studies were focused on explicating the adverse health and mental health impacts of foreclosure, three studies based on individual-level data also identified poor health as a determinant of foreclosure. Among the 174 study participants interviewed by Pollack and Lynch [29], most identified unemployment as the primary precipitant of foreclosure, but struggling to pay for costs associated with illness or hospitalization was the third most commonly named reason for foreclosure. In the two qualitative studies by Keene and colleagues [30], and Fields and colleagues [31] and Libman and colleagues [32], a common theme that emerged from the focus groups and in-depth interviews was a context of ongoing economic strain and vulnerability in which medical illness could trigger a cascade of troubles ultimately leading to foreclosure.

## Discussion

In this systematic review of the literature on foreclosure and health, most of the 35 identified studies showed that foreclosure has adverse effects on health, mental health, and health behaviors. At the individual level, the stress of personally experiencing foreclosure was associated with worsened mental health and adverse health behaviors, which were in turn linked to poorer health status. Similar mechanisms were elaborated through in-depth interviews in the qualitative studies reviewed. Studies based on multilevel data, while few in number, suggested that degradation of the neighborhood environment and increase in community stressors also had indirect, cross-level adverse effects on health and mental health. Finally, poor health was also identified as a cause of foreclosure, suggesting a possible mechanism for repeated and/or sustained exposures.

Interpretation of my findings is subject to several important limitations. First, as with all systematic reviews, I may have missed some studies, which would cause me to underestimate the extent of the literature on the adverse health and mental health impacts of foreclosure. It is also well known that qualitative studies can be difficult to locate using conventional search strategies [33]. However, I attempted to mitigate these possibilities by searching two bibliographic databases using a purposefully broad search protocol [34,35,36]. Second, there was considerable heterogeneity in the types of exposures and outcomes used, precluding a formal meta-analysis. The simple vote counting-styled procedure I employed to summarize my findings are characterized by low statistical power [37] and cannot assess the magnitude of the purported association. Nonetheless, the overall bent of the literature is fairly clear. Third, as previously noted, I excluded studies focused exclusively on earlier segments of the foreclosure process, such as mortgage delinquency or overall indebtedness or housing unaffordability. These studies generally yielded similar findings to those focused on foreclosure [38,39,40,41,42,43,44,45,46,47,48]. Therefore, it is highly unlikely that including them in my review would have altered my primary conclusions. Fourth, also as previously noted, I excluded studies about foreclosure, neighborhood degradation, and crime. These studies are principally drawn from the economics and sociology literature and are focused on testing sociological theories of disorder [49,50] or contagion effects of foreclosure on housing prices [51,52]. Finally, it is possible that publication bias may have affected the conclusions of my review. Unfortunately, methodological differences in the studies precluded the generation of summary measures that could permit such an analysis.

Most of the studies identified in this review were assessed to be at risk of bias, suggesting that more research is needed before a definitive causal association between foreclosures and health can be asserted. Despite this important limitation and the other limitations noted above, this systematic review suggests that, although the benefits of foreclosure prevention initiatives have generally been justified in economic terms [53,54], they can also have important health and mental health benefits as well. With an estimated five percent of U.S. residential properties currently more than 90 days past due or in the process of foreclosure [55], the U.S. foreclosure crisis has not yet run its course. Therefore, the potential implications of my findings for population health and mental health are substantial. The Obama administration’s signature response to the U.S. foreclosure crisis, the Making Home Affordable initiative, has provided assistance to millions of U.S. homeowners, most notably more than 1.4 million homeowners who have received permanent mortgage modifications under the Home Affordable Modification Program [56]. Yet the administration’s policy response was not launched until the U.S. foreclosure crisis had already reached its peak in 2009 [2]. Perhaps due to its delayed implementation [57], the Home Affordable Modification Program was estimated to have prevented only 800,000 foreclosures during its period of operation [58], far fewer than its anticipated target.

Other types of policy interventions, if provided early enough during the foreclosure process, may have beneficial spillover impacts on averting home foreclosure even if foreclosure reduction is not their intended outcome. Early intervention may be able to prevent an *acute* economic shock (such as that resulting from job loss or severe illness) from eventually developing into the *chronic* stress of foreclosure, with all of the attendant benefits this implies for health and mental health status [59,60,61]. Indeed, one recent study estimated that federal extensions of unemployment insurance—through the Extended Benefits, Emergency Unemployment Compensation, and Federal Additional Compensation programs—averted a total of 1.4 million foreclosures between 2008 and 2012 [62]. While an analysis employing quasi-experimental methods would be needed to provide empirical backing for making such policy recommendations on the grounds that they could improve health, the evidence presented in this review about the adverse health effects of foreclosure suggest that programs designed to encourage early return of foreclosed properties back into productive use may have health and mental health benefits.

Although I have reviewed a fairly extensive literature on foreclosure, health, and mental health (the bulk of which has been published in the past five years), several priority questions remain. First, few studies employed methods designed to permit inference about the extent to which the association between foreclosure and health and mental health is causal. The strongest study designs employed time and geographic unit (e.g., county) fixed-effects to adjust for potential confounding. There remains the possibility, of course, that findings in the aggregate may not transfer to individuals [63,64]. Related to the above, given that poor health has frequently been cited as a cause of foreclosure [29,31,32,65,66,67], it is possible that the observed association may be explained by reverse causality. Only two of the studies based on individual-level data accounted for potential selection effects by adjusting for baseline (pre-foreclosure) comorbidity. Most of the studies included in this review were based on data collected during a time period when foreclosures were disproportionately driven by plausibly exogenous factors such as declining housing prices [1]. However, it is also important to note that many studies did not perfectly overlap in timing with the Great Recession because the foreclosure rate began increasing prior to 2007, especially in the subprime market, and the foreclosure rate has remained elevated even after the Great Recession was officially pronounced to have run its course. Third, few studies provided opportunities to investigate the ways in which the adverse effects of foreclosure were exacerbated by other stressful life events or chronic strains, such as job loss or relationship dissolution. (Analogous studies using aggregate-level data would have examined, for example, interactions between rates of foreclosure and rates of unemployment.) A better understanding of other stressful life events or chronic strains can interact with the experience of foreclosure may suggest important levers that can be targeted through policy or program development. Fourth, all but five studies were based on data collected solely at either the individual or the aggregate level. Multilevel studies, which offer the ability to estimate cross-level effects [68,69], may allow for a richer understanding of how personal experience of foreclosure *and* exposure to proximate foreclosed properties may interact to shape the distribution of health and mental health.

It is notable that only two studies examined the extent to which foreclosure may have disproportionate impacts on ethnic or racial minority populations. This is an important gap in the literature because black and Latino families were more likely than any other ethnic or racial groups in the U.S. to have experienced foreclosure during the peak of the U.S. foreclosure crisis [70,71]. For black families specifically, these excess foreclosures have occurred in the context of decades of systematic, government-led policies designed to block the accumulation of wealth [15,72], which, while subsequently repealed, have provided fertile conditions for predatory subprime lending and reverse redlining [17,73,74]. While it may take decades for black and Latino families to recover wealth lost during the foreclosure crisis, the intergenerationally-compounded [75,76] adverse effects of foreclosure and racial and ethnic disparities in health are as of yet unknown.

## Supporting Information

S1 Checklist Preferred Reporting Items for Systematic Reviews and Meta-Analyses (PRISMA) Checklist. (DOCX)

S1 Table Search terms applied to PubMed and PsycINFO. (DOCX)

S2 Table Quality assessment for quantitative studies. (DOCX)
