# Supplementary material for: Home Foreclosure, Health, and Mental Health: A Systematic Review of Individual, Aggregate, and Contextual Associations
Source: PLoS One. 2015 Apr 7;10(4):e0123182. doi: 10.1371/journal.pone.0123182 (PMC4388711; doi:10.1371/journal.pone.0123182)
Supplement: S1 Table — (DOCX) [file pone.0123182.s002.docx]

**Table S1. Search terms applied to PubMed and PsycINFO**

| **Search Terms** |
| --- |
| *PubMed*  (bankruptcy[MH] OR foreclos*[TIAB] OR mortgag*[TIAB] OR bankrupt*[TIAB] OR "housing loss"[TIAB] OR "housing instability"[TIAB] OR "housing insecurity"[TIAB] OR "housing strain"[TIAB]) AND (mental disorders[MH] OR adjustment disorders[MH] OR anxiety disorders[MH] OR stress disorders, traumatic[MH] OR stress disorders, post-traumatic[MH] OR mood disorders[MH] OR depressive disorder[MH] OR depression, postpartum[MH] OR depressive disorder, major[MH] OR depressive disorder, treatment-resistant[MH] OR dysthymic disorder[MH] OR substance-related disorders[MH] OR alcohol-related disorders[MH] OR alcoholism[MH] or binge drinking[MH] OR drinking behavior[MH] OR alcohol drinking[MH] OR tobacco use[MH] OR smoking[MH] OR behavioral symptoms[MH] OR affective symptoms[MH] OR depression[MH] OR mental fatigue[MH] OR self-injurious behavior[MH] OR suicide[MH] OR suicidal ideation[MH] OR suicide, attempted[MH] OR stress, psychological[MH] OR violence[MH] OR domestic violence[MH] OR spouse abuse[MH] OR child abuse[MH] OR battered child syndrome[MH] OR child abuse, sexual[MH] OR ambulatory care[MH] OR hospitalization[MH] OR patient admission[MH] OR emergency medical services[MH] OR emergency services, psychiatric[MH] OR mental health services[MH] OR chronic disease[MH] OR "hypertension"[MH] OR body weight[MH] OR body weight changes[MH] OR weight gain[MH] OR obesity[MH] OR obesity, abdominal[MH] OR obesity, morbid[MH] OR overweight[MH] OR mental[TIAB] OR anxiety[TIAB] OR depress*[TIAB] OR alcohol*[TIAB] OR drinking[TIAB] OR "substance use"[TIAB] OR "substance abuse"[TIAB] OR drug OR smoking[TIAB] OR binge[TIAB] OR suicide[TIAB] OR stress[TIAB] OR distress[TIAB] OR "domestic violence"[TIAB] OR "partner violence"[TIAB] OR utiliz*[TIAB] OR emergency*[TIAB] OR hospitaliz*[TIAB] OR chronic[TIAB] OR hypertens*[TIAB] OR obes*[TIAB] OR weight[TIAB]) |
| *PsycINFO*  (TI (foreclos* OR mortgag* OR bankrupt* OR "housing loss" OR "housing instability" OR "housing insecurity" OR "housing strain") OR AB (foreclos* OR mortgag* OR bankrupt* OR "housing loss" OR "housing instability" OR "housing insecurity" OR "housing strain")) AND ((DE ("mental disorders" OR "chronic mental illness" OR "adjustment disorders" OR "posttraumatic stress disorder" OR "acute stress disorder" OR "stress reactions" OR "anxiety"" OR "anxiety disorders" OR "affective disorders" OR "major depression" OR "reactive depression" OR "recurrent depression" OR "treatment resistant depression" OR "depression (emotion)" OR "dysthymic disorder" OR "behavior disorders" OR "drug abuse" OR "alcohol abuse" OR "alcoholism" OR "binge drinking" OR "alcohol intoxication" OR "drug dependency" OR "inhalant abuse" OR "polydrug abuse" OR addiction OR "drug addiction" OR "heroin addiction" OR "self destructive behavior" OR "attempted suicide" OR "self injurious behavior" OR "suicide" OR "stress" OR "chronic stress" OR "financial strain" OR "psychological stress" OR "social stress" OR "stress reactions" OR "violence" OR "domestic violence" OR "intimate partner violence" OR "physical abuse" OR "partner abuse" OR "battered females" OR "child abuse" OR "outpatient treatment" OR "psychiatric clinics" OR "hospitalization" OR "psychiatric hospitalization" OR "psychiatric hospital admission" OR "mental health services" OR "chronic illness" OR "body weight OR "weight gain" OR "overweight" OR "obesity")) OR (TI (mental OR anxiety OR depress* OR alcohol* OR drinking OR "substance use" OR "substance abuse" OR drug OR smoking OR binge OR suicide OR stress OR distress OR "domestic violence" OR "partner violence" OR abuse OR utiliz* OR emergency* OR hospitaliz* OR chronic OR hypertens* OR obes* OR weight)) OR (AB (mental OR anxiety OR depress* OR alcohol* OR drinking OR "substance use" OR "substance abuse" OR drug OR smoking OR binge OR suicide OR stress OR distress OR "domestic violence" OR "partner violence" OR abuse OR utiliz* OR emergency* OR hospitaliz* OR chronic OR hypertens* OR obes* OR weight))) |
