# Supplementary material for: Home Foreclosure, Health, and Mental Health: A Systematic Review of Individual, Aggregate, and Contextual Associations
Source: PLoS One. 2015 Apr 7;10(4):e0123182. doi: 10.1371/journal.pone.0123182 (PMC4388711; doi:10.1371/journal.pone.0123182)
Supplement: S2 Table — (DOCX) [file pone.0123182.s003.docx]

**Table S2. Quality assessment for quantitative studies**

| **Citation** | **Population-Based** | **Objectively Measured Exposure** | **Objectively Measured Outcome** | **Exposure Precedes Outcome** | **Accounts for Unobserved Confounding** | **Risk of Bias** |
| --- | --- | --- | --- | --- | --- | --- |
| Arcaya and colleagues [77, 78] | Yes | Yes | Yes | Yes | No | At risk |
| Ayers and colleagues [79] | Yes | Yes | Yes | Yes | No | At risk |
| Batson and Monnat [80] | Yes | Yes | No | Yes | No | At risk |
| Brooks-Gunn and colleagues [81] | Yes | Yes | No | No | No | At risk |
| Burgard and colleagues [82] | Yes | No | No | No | No | At risk |
| Cagney and colleagues [83] | Yes | Yes | No | Yes | No | At risk |
| Cannuscio and colleagues [84] | No | No | No | No | No | At risk |
| Collier-Goubil [85] | Yes | Yes | Yes | No | No | At risk |
| Cook and Davis [65] | No | Yes | Yes | Yes | No | At risk |
| Currie and Tekin [86] | Yes | Yes | Yes | Yes | Yes | Low risk |
| Fowler and colleagues [88] | Yes | Yes | No | Yes | No | At risk |
| Freeman and colleagues [89] | Yes | No | No | No | No | At risk |
| Frioux and colleagues [90] | Yes | Yes | Yes | Yes | Yes | Low risk |
| Gili and colleagues [91] | No | No | Yes | No | No | At risk |
| Houle [92] | Yes | Yes | No | Yes | Yes | At risk |
| Houle and Light [18] | Yes | Yes | Yes | No | Yes | At risk |
| McLaughlin and colleagues [93] | Yes | No | No | Yes | No | At risk |
| Menzel and colleagues [94] | No | Yes | Yes | No | No | At risk |
| Mulia and colleagues [95], Zemore and colleagues [96], Murphy and colleagues [97] | Yes | No | No | No | No | At risk |
| Osypuk and colleagues [102] | No | No | No | No | No | At risk |
| Pence and colleagues [100], Mugavero and colleagues [101] | No | No | Yes | No | No | At risk |
| Pevalin [103] | Yes | No | No | No | No | At risk |
| Pollack and colleagues [104] | No | Yes | No | Yes | No | At risk |
| Pollack and Lynch [29] | No | No | No | No | No | At risk |
| Ragins and colleagues [105] | No | No | No | No | No | At risk |
| Reisen and colleagues [106, 107] | No | No | Yes | No | No | At risk |
| Schootman and colleagues [109] | No | Yes | No | No | No | At risk |
| Scully and colleagues [110] | Yes | No | No | No | No | At risk |
| Wood and colleagues [112] | Yes | Yes | Yes | Yes | Yes | Low risk |
